# Induced Autologous Stem Cell Transplantation for Treatment of Rabbit Renal Interstitial Fibrosis

**DOI:** 10.1371/journal.pone.0083507

**Published:** 2013-12-18

**Authors:** Guang-Ping Ruan, Fan Xu, Zi-An Li, Guang-Xu Zhu, Rong-Qing Pang, Jin-Xiang Wang, Xue-Min Cai, Jie He, Xiang Yao, Guang-Hong Ruan, Xin-Ming Xu, Xing-Hua Pan

**Affiliations:** Stem Cell Engineering Laboratory of Yunnan Province, Kunming General Hospital of Chengdu Military Command, Kunming, China; Fondazione IRCCS Ospedale Maggiore Policlinico & Fondazione D’Amico per la Ricerca sulle Malattie Renali, Italy

## Abstract

**Introduction:**

Renal interstitial fibrosis (RIF) is a significant cause of end-stage renal failure. The goal of this study was to characterize the distribution of transplanted induced autologous stem cells in a rabbit model of renal interstitial fibrosis and evaluate its therapeutic efficacy for treatment of renal interstitial fibrosis.

**Methods:**

A rabbit model of renal interstitial fibrosis was established. Autologous fibroblasts were cultured, induced and labeled with green fluorescent protein (GFP). These labeled stem cells were transplanted into the renal artery of model animals at 8 weeks.

**Results:**

Eight weeks following transplantation of induced autologous stem cells, significant reductions (*P* < 0.05) were observed in serum creatinine (SCr) (14.8 ± 1.9 mmol/L to 10.1 ± 2.1 mmol/L) and blood urea nitrogen (BUN) (119 ± 22 µmol/L to 97 ± 13 µmol/L), indicating improvement in renal function.

**Conclusions:**

We successfully established a rabbit model of renal interstitial fibrosis and demonstrated that transplantation of induced autologous stem cells can repair kidney damage within 8 weeks. The repair occurred by both inhibition of further development of renal interstitial fibrosis and partial reversal of pre-existing renal interstitial fibrosis. These beneficial effects lead to the development of normal tissue structure and improved renal function.

## Introduction

Renal interstitial fibrosis (RIF) is a significant cause of end-stage renal failure. It can occur at different stages of intrinsic renal cell apoptosis, leading to tubular atrophy. Chronic and progressive renal functional insufficiency appears at the later stages of this pathological process. Patients typically receive renal replacement therapy as a lifelong treatment. There is no effective drug treatment for clinical RIF. Therefore, the inability to prevent or decrease progression and eventually reverse the occurrence and development of RIF is a global problem. Stem cells are a class of self-renewal and multilineage differentiation capacity cells; studies have reported that stem cells can differentiate into renal tubular epithelial cells [1], glomerular endothelial cells, mesangial cells and podocytes [2,3]. This differentiation is important for structural remodeling and functional regeneration of renal tissue [4].

Unilateral ureteral ligation is an established model of RIF [5,6]. Within two weeks of ligation, there is proliferation of fibroblasts and the formation of mesenchymal extracellular matrix. Inflammatory cells infiltrate the kidney tissue, leading to severe damage to the tubular and mesenchymal structure, and eventual fibrosis. However, there are virtually no lesions in the glomerulus. Therefore, this model is suitable for the study of renal interstitial fibrosis and development of potential anti-fibrosis treatments. In this study, the unilateral ureteral ligation method was used as a model of RIF.

Stem cells are a class of self-renewal cells with unlimited proliferation and multi-differentiation potential, and are divided into three classes: 1) The embryonic stem cell (ESC): These refer to the inner cell mass or primitive reproductive cells obtained by special *in vitro* culture methods and cell sorting. Prior studies have shown that ESCs can differentiate into kidney parenchymal cells. 2) Adult stem cells: These have ability to self-update; adult stem cells exist in a variety of tissues of mature individuals, such as hematopoietic stem cells (HSC), bone marrow mesenchymal stem cells (MSC), nerve stem cells (NSC), muscle stem cells, osteogenesis stem cells, endodermal stem cells and retinal stem cells. The most studied and widely used stem cells are those obtained from the bone marrow. Bone marrow includes at least two types of stem cells, hematopoietic stem cells (HSCs) and mesenchymal stem cells (MSCs). Mesenchymal stromal cells, originally described in the 1960s as bone-forming cells in the bone marrow, are now called multipotent mesenchymal stromal cells or more commonly MSCs because they display adult stem cell multipotency. Thus, they differentiate into bone, cartilage and other connective tissues [7]. These capabilities have significant implications for structural remodeling and functional regeneration of renal tissue. 3) Induced pluripotent stem cells [8]: These are somatic cells into which genes are transferred to make them capable of differentiation and proliferation. Specific small molecules can be added to the culture medium so that the somatic cells can be reprogrammed into pluripotent stem cells [9]. Somatic cell reprogramming overcomes the limited source of seed cells, immune rejection response, ethical concerns, and other traditional insurmountable obstacles to stem cell research policy, and has broad prospects for clinical application [10]. The use of induced pluripotent stem cells to treat kidney disease has not yet been reported. Opponents of stem-cell research have welcomed iPS-cell technology as a method for achieving an embryonic-like state without the ethical dilemma of destroying human embryos. Therefore, iPS-cell technology is especially attractive for researchers in countries in which the use of embryonic cells is restricted [11].

Mouse iPS cells have been differentiated into hematopoietic precursor cells and have been shown to rescue lethally-irradiated mice. Studies have reported the use of stem cells in treatment of selected kidney diseases [12], such as IgA nephropathy, chronic aristolochic acid nephropathy, starch deposition kidney disease, focal segmental glomerulosclerosis, rapidly progressive glomerulonephritis, lupus nephritis, acute and chronic renal failure, and end-stage kidney disease [3].

There are several innovative and significant aspects of this research. Stem cell research is of widespread interest at the frontiers of life sciences, but due to the shortage of sources, ethical concerns, immune rejection and other problems, the development of the discipline has been seriously restricted. Therefore, it is important to find new sources of stem cells for research. The current study exploits a natural inducer [13], using different doses and different induction times on various types of animal fibroblasts, to produce multipotent stem cells. After induction, morphological observation, immunohistochemical and PCR identification, and epigenetic and vaccination teratoma methods have been applied to use fibroblasts, under control of the inducer, to reverse differentiation and provide broad prospects for clinical research.

The goals of the present study are to take advantage of use of this inducer to characterize the effects of cell re-infusion in a rabbit model of RIF. These results demonstrate that the induced stem cells can promote differentiation of rabbit autologous fibroblasts to become induced multipotent stem cells and elicit a protective role in the treatment of RIF.

## Materials and Methods

### 1: Experimental animals

Thirty-five healthy Japanese white rabbits (aged 3 months), weighing 2.3 ± 0.3 kg, were provided by the Experimental Animal Center, Kunming General Hospital of Chengdu Military Command (certificate number: SYXK (Yunnan) 2005-2008; experimental facility conditions permit No.: 2007-041 SYXK (Army)). The two primary groups of rabbits were: normal group (n = 5) with no treatment and the model group (n = 30) with left ureteral ligation after 8 weeks. The model group was then randomly divided into three subgroups: 1) Transplantation of induced cell group (n = 10), which featured left ureteral ligation followed by transfusion of induced autologous skin fibroblasts; and 2) transplantation of un-induced cell group (n = 10), featuring left ureteral ligation followed by transfusion of the non-induced autologous skin fibroblasts; and 3) model rabbits without treatment (n = 10). Experimental protocols were approved by the Experimental Animal Ethics Committee of Kunming General Hospital of Chengdu Military Command.

### 2: Left ureteral ligation as an animal model of RIF

#### 2.1: Methods

Rabbits were anesthetized with 3% pentobarbital sodium (1 ml/kg) through the marginal ear vein. The surgical field was shaved, with the rabbit fixed on the surgical board in the right lateral position. The skin was routinely disinfected. On top of the left kidney, a 3-cm skin incision was made to expose the left kidney and ureter. Tissue forceps were used to hold the ureter in place. About 5 cm of the ureter was isolated, and each side was ligated by the surgical line. The kidney capsule and surrounding tissue was then separated. After surgery, surrounding muscle and skin were sutured. Three days following surgery, an intramuscular injection of penicillin was given to prevent infection at the site of incision.

#### 2.2: Biochemical detection

Before establishment of the RIF model, 1, 2, 4, and 8 weeks after ligating the ureter, and 1, 4, and 8 weeks after cell transplantation, 2 ml of blood was drawn from the rabbit marginal ear vein. A Hitachi-7171A automatic biochemical analyzer was used to determine SCr and BUN content.

#### 2.3: Single photon emission computer tomography apparatus (SPECT) monitoring

At 8 weeks after cell transplantation, animals in the four groups underwent radionuclide dynamic renal scanning, using the Discovery VH dual probe of SPECT (GE Company; Haifa, Israel) to produce nuclide renal dynamic imaging. The Kunming General Hospital Department of Nuclear Medicine assisted in these measurements.

#### 2.4: Renal gross morphology and left kidney weight observations

At 16 weeks (8 weeks after transplantation), animals from the four groups were randomly sacrificed using the ear vein injection of air embolism method under general anesthesia. The color and volume change of the kidney was noted.

### 3: Culture, transfection, and induction of fibroblasts

Approximately 2-3 cm^2^ of leg skin was removed under sterile conditions and placed in PBS containing 100 U/ml ampicillin and 100 mg/ml streptomycin sulfate for 10 min. The leg skin was rinsed again with PBS and subcutaneous fat and connective tissue were removed. The skin was cut into small pieces of about 1 mm^3^ in volume and 0.25% trypsin and 0.02% EDTA in PBS was added to the samples. Tissues were placed in a 37°C air shaker at 180 rpm for 20 min, 10% fetal bovine serum was added to media after 5 min, samples were centrifuged for 10 min at 300 g, the supernatant was removed, and Dulbecco’s Modified Eagle’s Medium / Ham’s F12 (DMEM/F12) media was used to wash the cells twice. DMEM containing 10% fetal bovine serum was added after the cells were inoculated in culture flasks. Cells were placed in 37°C, 5% CO_2_, and saturated humidity conditions for culture [7,8]. After 48 to 72 h, media were changed for the first time, and non-attached cells were discarded. Within 3 to 4 days, cells became a single layer and were subcultured using 0.25% trypsin. The eGFP PURO Lentivirus(1×10^9^ TU/ml)(from Genomeditech, Co., LTD, shanghai) was added for 48 h, media were changed, and the transfection efficiency was measured by flow cytometry. Inducer (fish oocyte extracts, developed in our laboratory) [13] was added to cells at a final concentration of 2 mg/ml, induction proceeded for 72 h. The cells were collected and identified by flow cytometry and q-PCR. The results showed the induced cells expressed stem cell markers of Oct-3/4, Nanog, SSEA-4.Then the cell density was adjusted to 2 × 10^5^ cells/ml for transplantation.

### 4: Detection of GFP transfection by flow cytometry

After transfection, cells were digested with 0.25% trypsin, centrifuged at 500 g for 3 min, the supernatant was removed, and 10 ml DMEM containing 10% fetal calf serum was added. After repeated washing, cells were centrifuged at 500 g for 3 min, supernatant was removed, and a 1-ml single cell suspension was made. An aliquot of the cell suspension (0.5 ml) was used for flow cytometry (Becton Dickinson; Frankin Lakes, NJ, USA) to determine GFP transfection efficiency. Untransfected cells were used as a negative control.

### 5: Collection and identification of induced skin fibroblasts

After induction, cells were collected and done flow cytometry and quantitative PCR to detect stem cell markers, including SSEA-4, Oct-3/4 and Nanog. Induced and non-induced cells were digested with trypsin, and digestion was terminated by adding serum-containing medium. The cell suspension was transferred to a centrifuge tube and centrifuged at 500 g for 4 min, the supernatant was removed, and cells were washed three times with normal saline. Cells were collected at a concentration of 2 × 10^5^ cells/ml.

### 6: Cell transplantation

Rabbits were laid supine on the operating table, were anesthetized with an ear vein injection of 1 ml of 3% pentobarbital sodium per kg body weight. Hair was removed from the upper abdomen and left groin area, and the shaved area was disinfected. An incision was made in the left groin area to expose the left femoral artery. Artery clips were placed at the proximal and distal ends of the femoral artery. Ophthalmic scissors were used to cut a small port in the arterial wall between the two artery clips. The catheter for anesthesia and syringe were moistened with heparinized saline, and the catheter was inserted into the left renal artery and abdominal aortic bifurcation approximately 1 cm below the bifurcation of the left renal artery and abdominal aorta. The catheter was inserted approximately 1 cm into the abdominal aorta in preparation for cell transplantation. Animals assigned to the induced cell group were injected with induced cells (2×10^5^ cells/ml), and those assigned to the non-induced group were injected with non-induced cells (2×10^5^ cells/ml). It took about 1 min to inject the cells into the renal artery. Following injection, the puncture site near the heart-side of the arterial clip was clamped, and the blood vessel wall was sutured to prevent cell washout. Penicillin (800,000 U) was injected into the abdominal cavity to prevent infection, the abdomen was closed, and the surface was disinfected.

### 7: SPECT to check the renal glomerular filtration rate

At 8 weeks after transplantation, three rabbits were randomly selected from each of the four groups. Anesthesia was induced by an ear vein injection of 1 ml of 3% pentobarbital sodium per kg body weight. A bolus dose of 3.5 mCi of ^99^mTc-DTPA (diethylenetriamine pentaacetic acid) in 0.2 ml was administered through the ear vein. A total of 30 renal perfusion images were dynamically collected at 0.5-s intervals and a total of 20 images were collected at 1-min intervals to calculate the renal glomerular filtration rate (GFR) (ml/min). Entegra software (GE Corporation; Haifa, Israel) was used to process the acquired data. Kidney function was tested and compared among the different groups of animals. Through the use of SPECT, the renal GFR (in ml/min) can be calculated, thereby providing an assessment of kidney function.

### 8: Transforming growth factor (TGF-β1) immunohistochemistry assay

At 8 weeks after transplantation, 2-µm thick, paraffin-embedded sections of kidney tissues were prepared with conventional dewaxing rehydration. Sections were treated with Triton X-100 (0.1%) at room temperature for 10 min, then incubated with a 3% hydrogen peroxide - methanol solution for 15 min at 37°C, and finally incubated for 15 min with10% goat serum. Tissues were then exposed to a polyclonal rabbit anti-TGF-β1 antibody (from Wuhan Boster Company; Wuhan, China) at 4°C overnight. The secondary antibody was added to the tissues and the samples were incubated at 37°C for 30 min. Samples were stained with diaminobenzidine (DAB) and hematoxylin and then mounted with neutral gum after xylene dehydration. The tawny-colored tissue was identified as positive staining for TGF-β1. Image Proplus multimedia color pathological image software was used to analyze the results. Twenty non-overlapping fields at 200X magnification were assessed for each sample. The ratio of positive staining area within the field of vision to total tubulointerstitial area (after removal of the tubular lumen space) was calculated and averaged.

### 9: Data analysis

Experimental data are expressed as the mean ± SD (_x ± s). SPSS 17.0 software was used for analysis of variance and determination of statistical differences in serum renal function parameters. Treatment effects among the four groups of samples were compared using a one-way or two-way ANOVA or ANOVA for repeated measurement. Statistically significant differences were identified when *P* < 0.05.

## Results

### 1: Morphology

**Figure 1:**
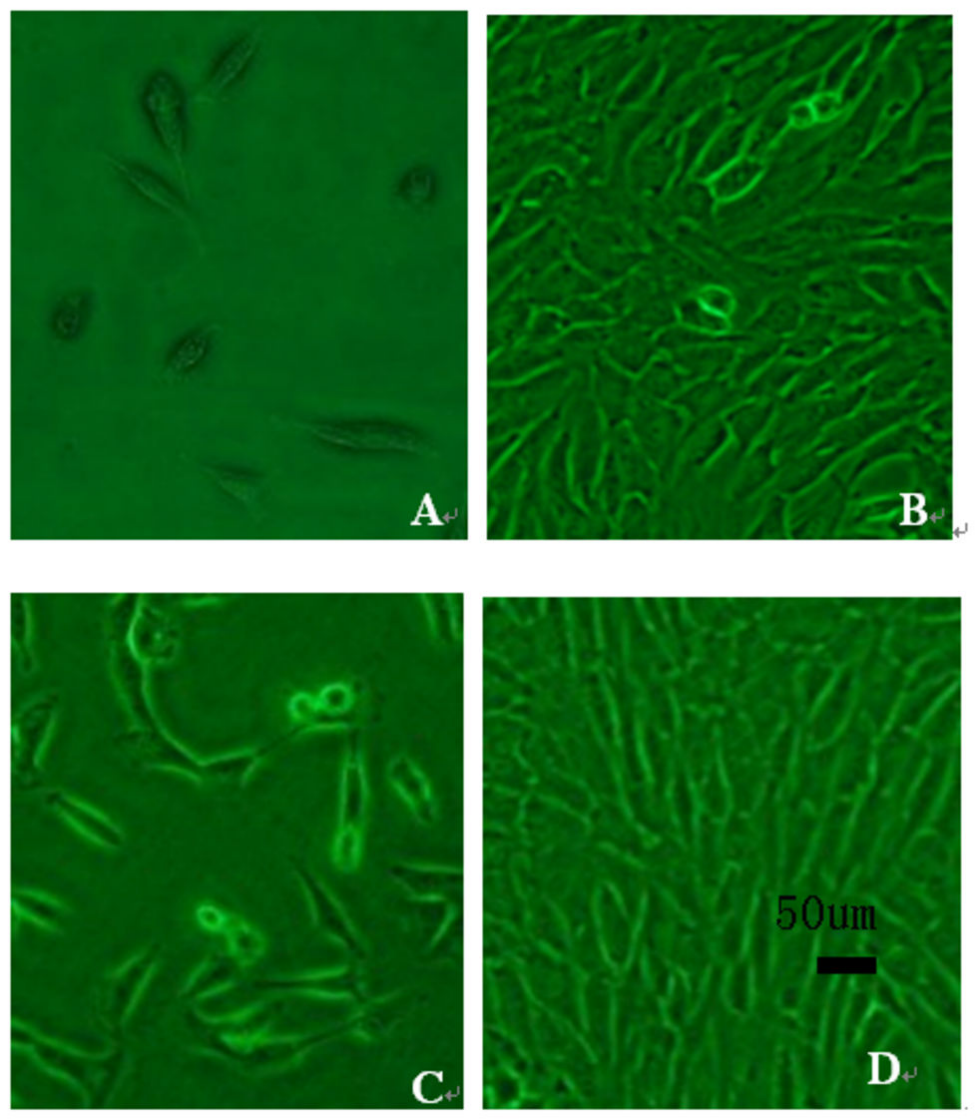
Morphology of rabbit fibroblasts (× 200). A. Primary culture for 2 days. B. Primary culture to 80% confluence. C. 1:1 passage after 2 days. D. Passaged cells cultured to 80% confluence.

**Figure 2:**
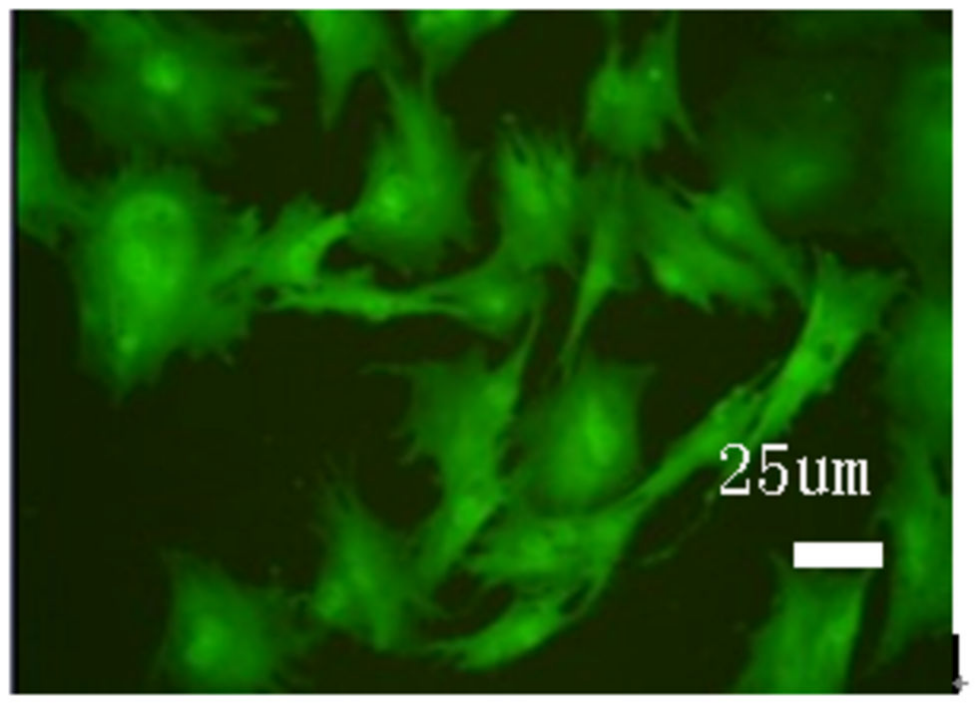
GFP-labeled fibroblasts (× 400). Cells were labeled with green fluorescent protein (GFP).

**Figure 3:**
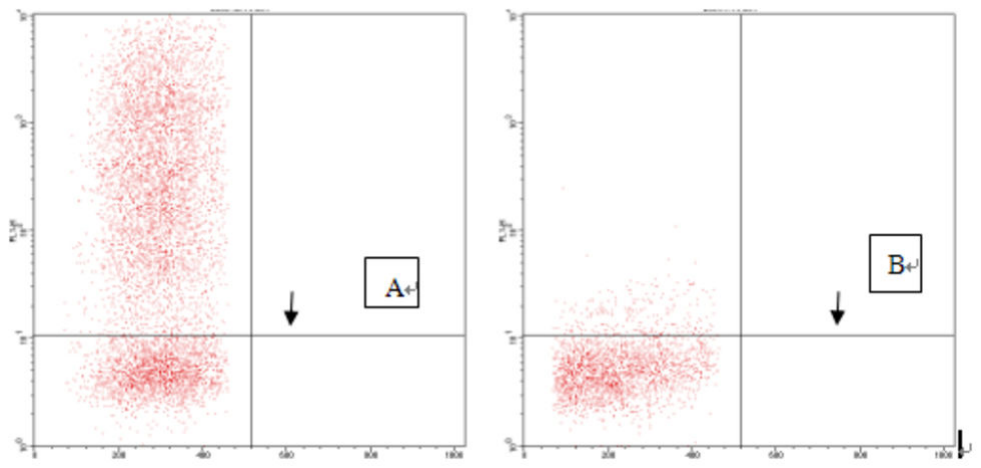
Monitoring of transfection rate in GFP-labeled cells. A. Positive green fluorescent protein (GFP)-labeled cells. B. Negative control of untransfected cells.

**Figure 4:**
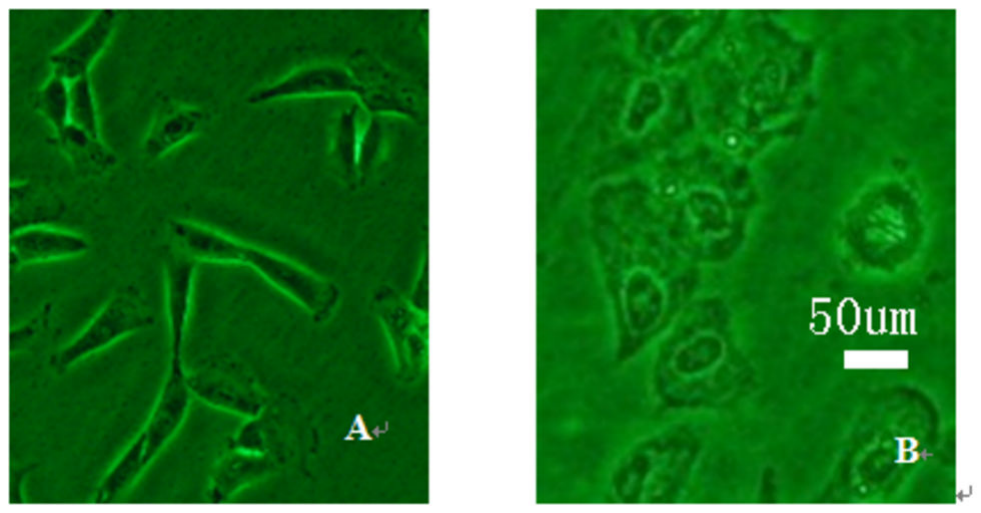
Morphological changes in induced processes. A. Non-induced cells were spindle-shaped (× 200). B. Induced cells were irregularly shaped and size became larger (× 200).

**Figure 5:**
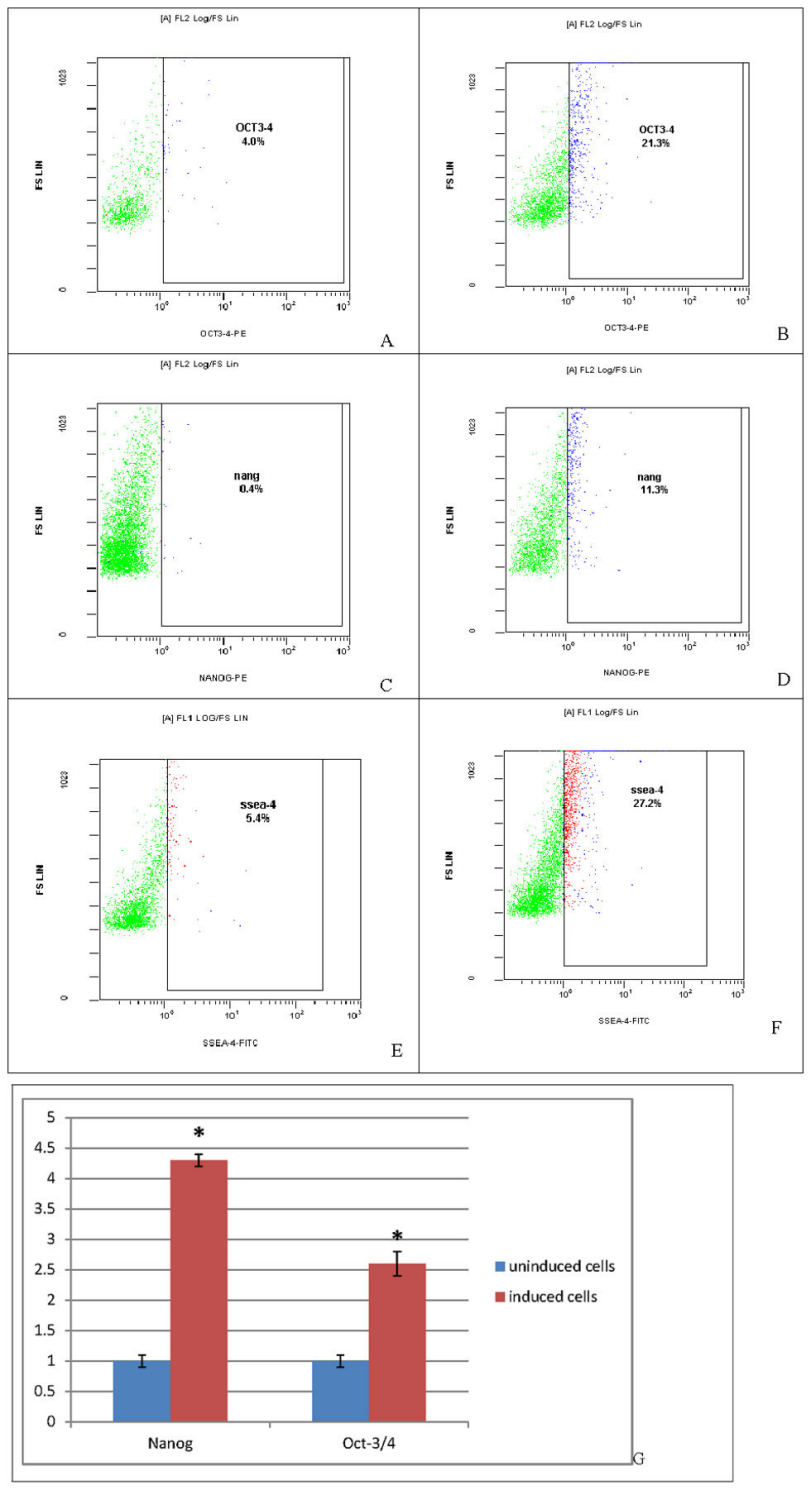
Identification of induced cells. A-F. Flow cytometry analysis revealed that the positive rates of Oct-3/4 and Nanog and SSEA-4 expressions in induced cells were 21.3, 11.3, and 27.2%, in non-induced cells were 4, 0.4, and 5.4%. The difference was statistically significant.. G. Quantitative PCR results showed the Oct-3/4 and Nanog genes expressions were increased significantly in induced cells compared to non-induced cells(* *P*<0.05).

Cultured fibroblasts were inoculated in 75 cm^2^ flasks and grown for 1 to 2 days. Adherent cells were spindle-shaped, and 3 to 4 days were required to achieve confluence. Passaged cells were uniformly distributed, and 1 to 2 days were required to achieve confluence (Figure 1). After isolation and culture of adherent fibroblasts, cells were stably passaged and observed under a fluorescence microscope 72 h after GFP transfection (Figure 2). Transfection rate was determined by flow cytometry (Figure 3). Figure 3A shows that the transfection rate was 80%. Uninduced cells were long and spindle-shaped, as shown in Figure 4A. After induction, cell morphology was characterized by a change from spindle-shaped to a larger, irregular shape (Figure 4B). Flow cytometry analysis revealed that the positive rates of Oct-3/4 and Nanog and SSEA-4 expressions in induced cells were 21.3, 11.3, 27.2%, in non-induced cells were 4, 0.4, 5.4%. The difference was statistically significant. Figure 5A-F). Quantitative PCR results showed the Oct-3/4 and Nanog genes expressions were increased significantly in induced cells(* *P*<0.05) compared to non-induced cells(Figure 5G).

### 2: General condition of animals

Model rabbits gradually decreased their level of activity after surgery. Hair was disheveled and dull and consumption of both food and drinking water was decreased. The normal control group of rabbits exhibited typical behavior, alert responses, shiny hair, and normal food intake. Compared with the model group, the difference in food intake was significant (*P* < 0.05), but there was no significant difference in body weight between the induced group and non-induced group.

### 3: Gross observation of kidneys and comparison of left kidney weights

**Figure 6:**
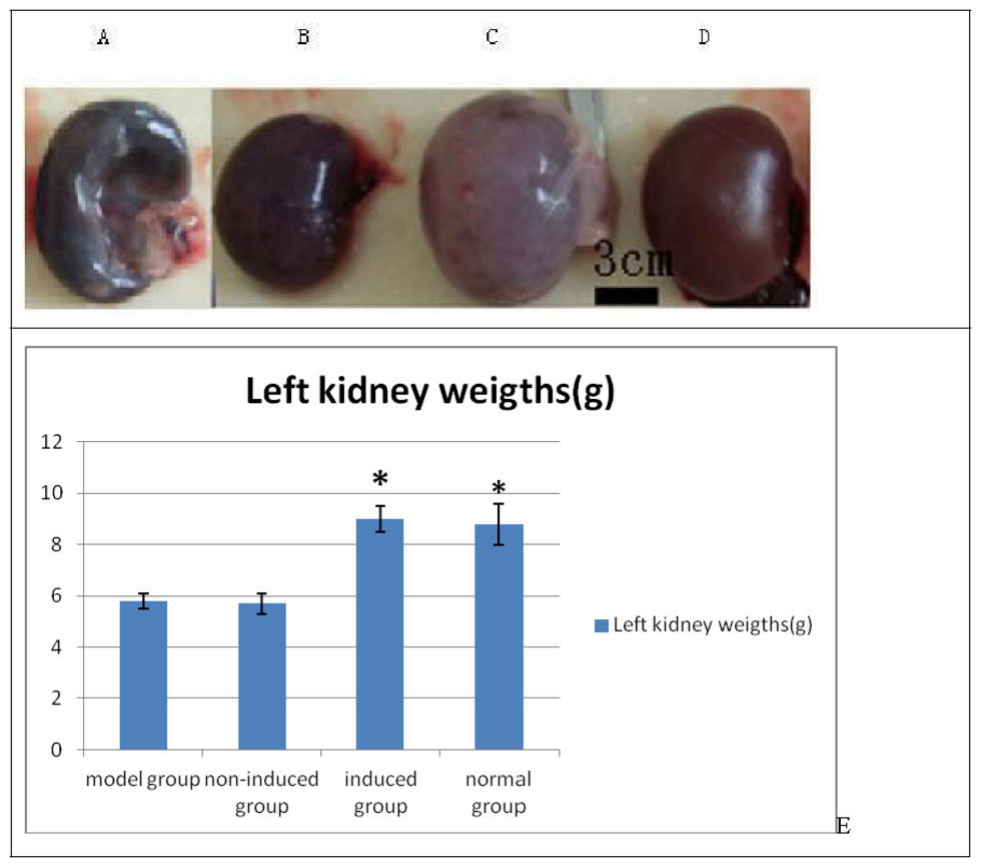
Left kidney specimens at 8 weeks after transplant. A. Model group. B. Non-induced group. C. Induced group. D. Normal group. E. Comparison of left kidney weight (n = 3). A one-way ANOVA revealed significant differences among groups (*P* < 0.01). The left kidney weights of the model and non-induced groups were significantly less than those of the normal and induced groups (*P < 0.01).

At 8 weeks after cell transplantation, rabbits were euthanized by the air embolism method. There was no significant change in the red, shiny color of the left and right kidneys relative to those in the normal group of rabbits and there was no expansion of the ureter. The total volume of kidneys in the model and non-induced group was significantly less than normal, the kidneys exhibited a darker color, and the ureter was dilated and obstructed. The renal parenchyma was significantly thinner and cortical and medullary regions were poorly defined. In the induced group, kidney volume was slightly larger than or equal to the normal kidney volume, the kidneys were pale in color, and the renal parenchyma was thinner than that of the normal group (Figure 6). The left kidney weights of the four groups were compared (Figure 6). A one-way ANOVA was used for statistical analysis because the data have only one factor (group). The results of one-way ANOVA indicated *P* < 0.01 when compared among groups. The left kidney weights of the model and non-induced group were significantly less than those of the normal and induced group (*P* < 0.01). In contrast, there was no significant difference (*P* = 0.873) between the left kidney weights of the model and non-induced group or those of the normal and induced group (*P* = 0.692).

### 4: Whole left kidney section analysis

**Figure 7:**
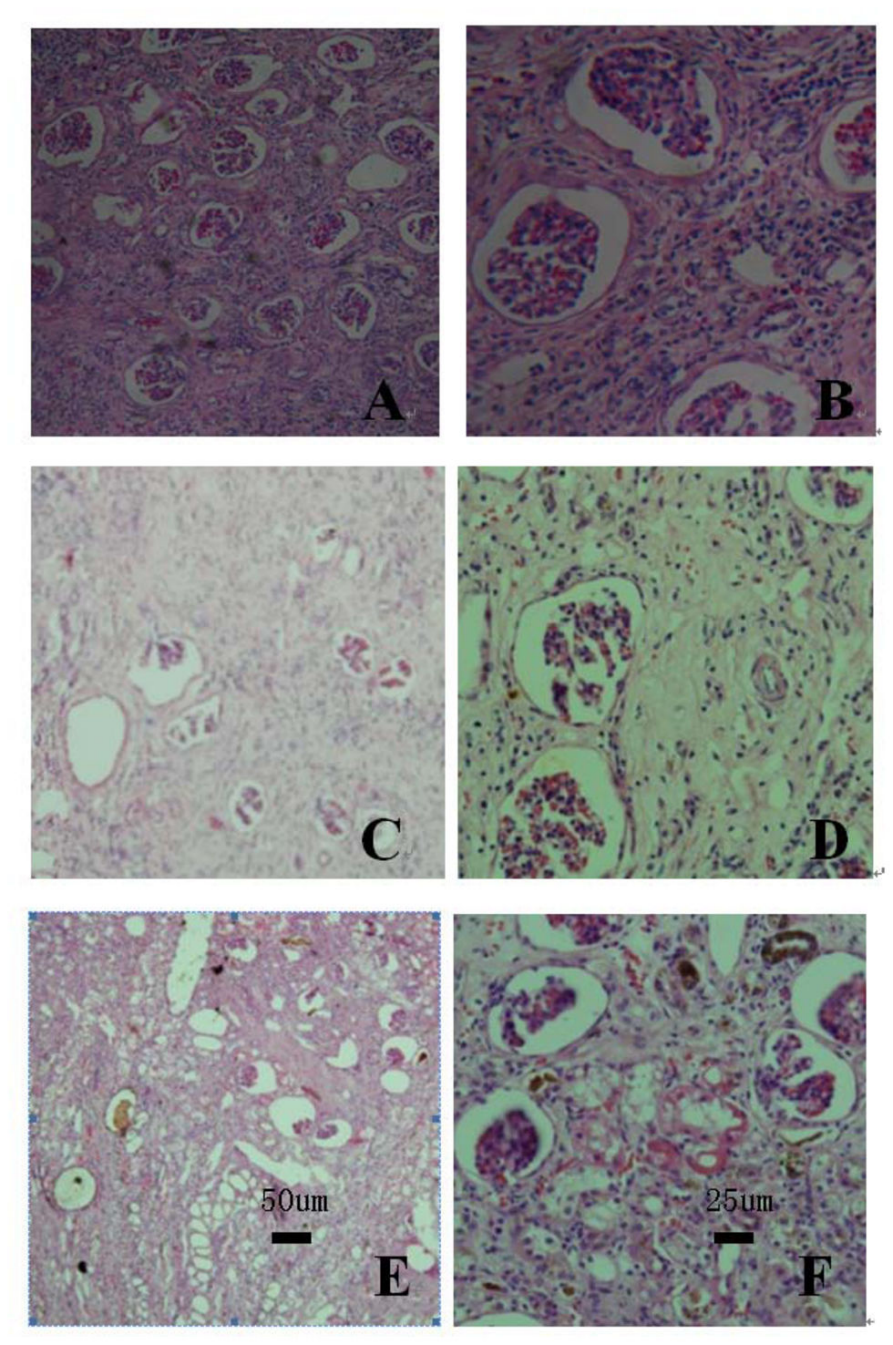
Morphological structure of left kidneys at 8 weeks after transplant. A, C, E are hematoxylin-eosin (HE) staining of renal tissue from the normal group, non-induced group and induced group, respectively (× 200). B, D, F are HE staining of renal tissue from the normal group, non-induced group and induced group, respectively (× 400).

Renal histopathology in the normal group showed no proliferation or sediment in the mesangial area (Figure 7A and 7B). There was no thickening of the glomerular capillary wall, the vascular cavity was not narrow, and the vascular loop exhibited no necrosis. No lesions were detected in the basement membrane. The glomerular capsule had no adhesions or lesions. All levels of renal tubules and collecting ducts exhibited normal structure. The renal tubular and collecting duct lumens did not exhibit a tube-like shape. Renal histopathology in the model and non-induced group showed reduction in the number of renal tubules (Figure 7C and 7D). The renal tubular basement membrane increased in thickness and exhibited structural disorder. Expansion of glomerular cysts and occlusion of the intracavity capillary ball was consistent with ischemic changes. Diffuse infiltration of inflammatory cells and severe fibrosis were observed in the renal interstitium. The induced group exhibited moderate renal tubular atrophy with partial thickening of the basement membrane, multifocal expansion of the tubules, and expansion of glomerular cysts (Figure 7E and 7F). Diffuse infiltration of inflammatory cells and mild fibrosis were observed in the renal interstitium.

The cause of inflammation is likely to be the disease model itself, and not a side effect of autologous cell transplantation. This is because the number of inflammatory cells decreased after transplantation. Ten sections of the left kidney in each group and ten visual fields from each section were analyzed. In total, therefore, one hundred visual fields from each group were analyzed and the results are summarized in Table 1. The independent variables are groups and different changes, whereas the dependent variable is the number of fields of pathological changes among the 100 visual fields. Accordingly, a two-way ANOVA was used for statistical analysis. The results of two-way ANOVA indicated *P* < 0.01 when comparing the number of fields of pathological changes among groups. Multiple comparison results showed the changes between the model and non-induced group were not significantly different (*P* > 0.05), but the changes between any other two pairs of groups was significantly different (*P* < 0.01).

**Table 1:** Analysis of one hundred visual fields of each group for pathological changes.

Pathological changes	Normal group*	Model group*	Non-induced group	Induced group*
Proliferation in the mesangial area	3	35	36	12
Thickening of the glomerular capillary wall	3	34	32	15
The vascular cavity was narrow	4	31	30	16
The vascular loop exhibited necrosis	2	29	30	14
Lesions in the basement membrane	2	30	32	15
The glomerular capsule had adhesions or lesions	1	28	29	12
Reduction in the number of renal tubules	0	29	27	10
Diffuse infiltration of inflammatory cells	1	28	26	11
Fibrosis in the renal interstitium	2	31	33	15
Thickening of the basement membrane	2	39	38	12
Expansion of glomerular cysts	0	40	41	16

A two-way ANOVA revealed significant differences among groups (*P < 0.01). There are significant differences between any two groups except between model and non-induced group.

### 5: Biochemical parameters in blood from animals in each group at different time points

After RIF modeling for 0, 1, 4, 8, 9, 12, and 16 weeks (Note: Cell transplantion was performed at 8 weeks after establishment of the model), changes in blood biochemical indicators were measured (Tables 2 and 3). At 8 weeks after transplantation, SCr values were significantly lower in the induced group than in the non-induced group and the model group, but were still higher than those in the normal group. In this case, ANOVA for repeated measurement method was used for statistical analysis. The ANOVA for repeated measurement analysis for SCr and BUN indicated *P* < 0.05 when values were compared among groups. 

**Table 2:** Serum creatinine ((x±s) mmol/l) levels before and after modeling and transplantation.

Groups		*N*	0 w	1 w	4 w	8 w	9 w	12 w	16 w
Normal group*		5	8.54 ± 1.37	8.84 ± 1.55	8.64 ± 1.06	9.78 ± 1.26	9.20 ± 1.58	8.26 ± 1.11	7.70 ± 1.83
Model group*		10	9.40 ± 1.13	10.1 ± 1.1	12.9 ± 1.4	14.1 ± 1.9	15.9 ± 2.1	15.1 ± 1.9	18.5 ± 3.1
Non-induced group*	10	9.30 ± 1.27	10.6 ± 0.5	12.8 ± 1.5	14.9 ± 2.1	15.1 ± 2.2	15.4 ± 2.3	17.7 ± 3.2	
Induced group		10	6.72 ± 0.95	9.11 ± 1.73	13.1 ± 1.1	14.8 ± 1.9	12.9 ± 2.1	11.9 ± 2.0	10.1 ± 2.1

Note: The results of ANOVA for repeated measurement indicated *P* < 0.05 when values were compared among groups. There are significant differences between normal and model groups (*P< 0.05), between normal and non-induced groups (*P< 0.05), but there are no significant differences between other two groups.

**Table 3:** Blood urea nitrogen ((x±s) µmol/l) levels before and after modeling and transplantation.

Groups		*N*	0 w	1 w	4 w	8 w	9 w	12 w	16 w
Normal group*		5	96.6 ± 14.4	132 ± 10	91.0 ± 17.6	105 ± 16	97.0 ± 11.3	89.6 ± 14.1	87.8 ± 11.9
Model group*	10	99.5 ± 5.9	129 ± 8	152 ± 11	172 ± 17	187 ± 19	180 ± 18	205 ± 21	
Non-induced group**		10	99.6 ± 6.7	131 ± 9	149 ± 13	178 ± 18	178 ± 20	183 ± 26	202 ± 24
Induced group**		10	92.7 ± 8.6	95.2 ± 9.9	131 ± 13	119 ± 22	104 ± 21	99 ± 20	97 ± 13

Note: The results of ANOVA for repeated measurement indicated *P* < 0.05 when values were compared among groups. There are significant differences between any two groups (*, **P < 0.01) except between model and non-induced groups, and between normal and induced groups (*P* > 0.05).

### 6: SPECT detection results

**Figure 8:**
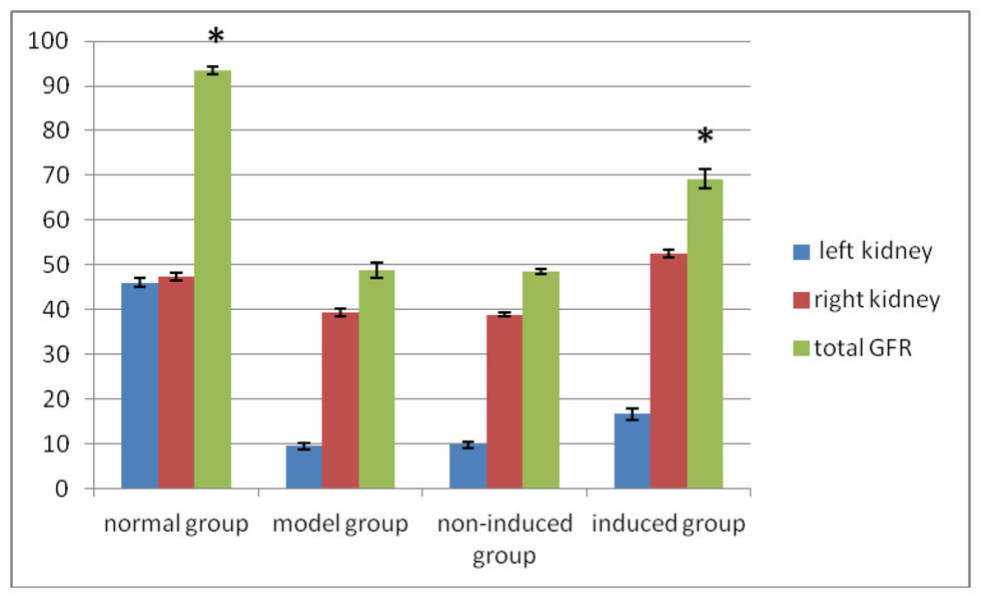
Renal glomerular filtration rate (GFR) (ml/min) at 8 weeks after cell transplantation (n = 3). A two-way ANOVA revealed significant differences among groups (*P<0.01).

**Figure 9:**
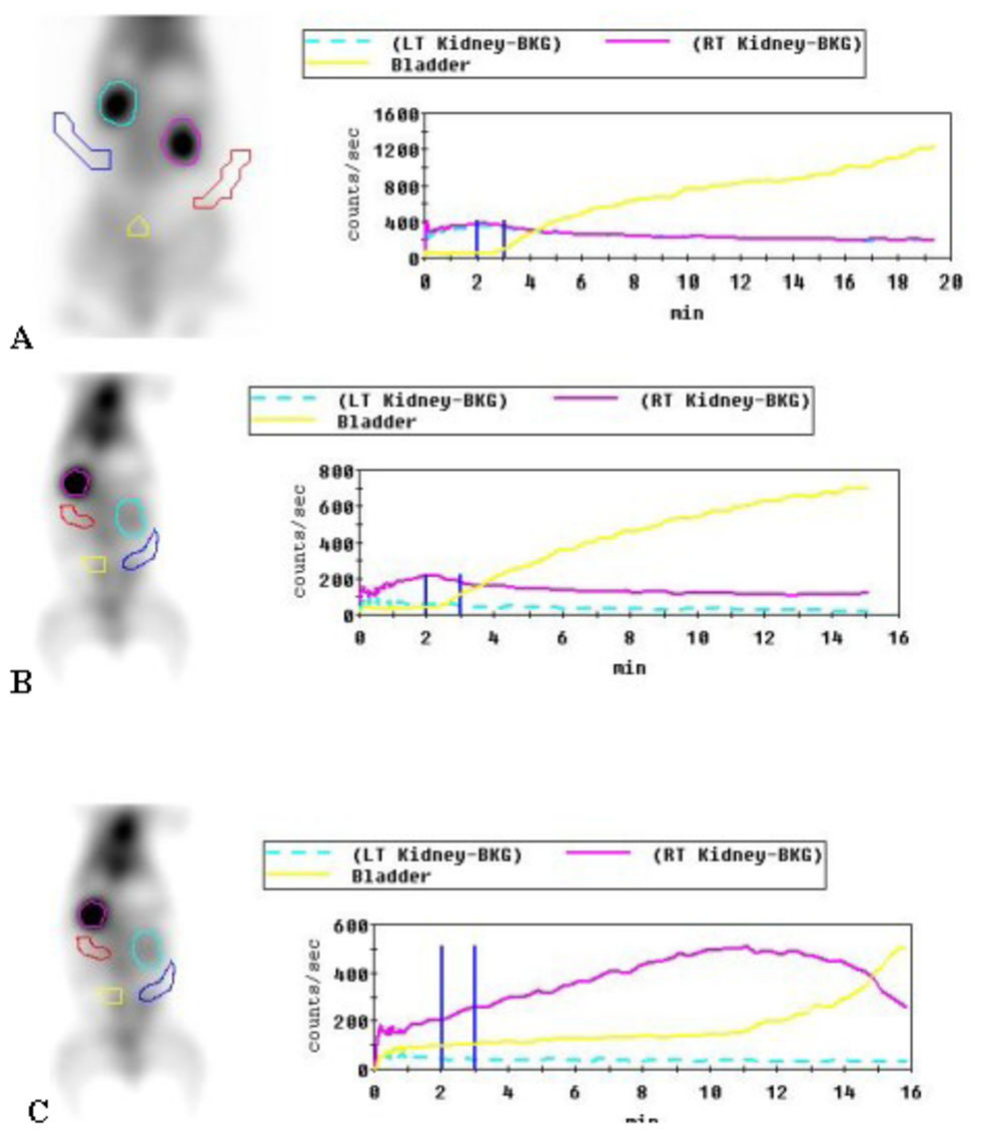
Single photon emission computer tomography (SPECT) analysis at 8 weeks after cell transplantation. Note: yellow indicates bladder urine volume with increasing time, the red line represents a relatively normal side of the renal uptake of contrast agent, and the green line represents the obstructed kidney uptake of contrast agent. A. Normal group; SPECT shows glomerular filtration rate. B. Non-induced group; SPECT shows decreased glomerular filtration rate. C. Induced group; SPECT shows significant improvement in glomerular filtration rate.

Ear vein bolus administration, SPECT analysis, Entegra software processing, and acquisition of data were conducted. All values reported are n = 3. In the normal control group, the value of GFR (ml/min) for the left kidney was 46 ± 1 and that for the right kidney was 47.4 ± 0.8, giving a total GFR of 93.4 ± 0.8. For the model group, the left kidney had a lower GFR value - the left kidney GFR (obstructed kidney) was 9.5 ± 0.7 and the right kidney GFR was 39.3 ± 0.9, giving a total GFR value of 48.8 ± 1.6. For the non-induced group, the left kidney had a lower GFR value - the left kidney GFR (obstructed kidney) was 9.8 ± 0.7 and the right kidney GFR was 38.8 ± 0.4, giving a total GFR value of 48.6 ± 0.6. The GFR of the obstructed kidney in the induced group was slightly higher than that for the non-induced group - the left kidney GFR was 16.7 ± 1.3 and the right kidney GFR was 52.5 ± 0.8, giving a total GFR value of 69.2 ± 2.1 (Figure 8). In this case, the independent variables are groups and right or left kidneys, the dependent variable is the GFR and thus, the two-way ANOVA method was used for statistical analysis. The results of two-way ANOVA indicated *P* < 0.01 when values were compared among groups. GFR for the model and non-induced group kidney decreased significantly, exhibiting almost a complete loss of function, while the GFR for the contralateral, normal right kidney also decreased, indicating that the right kidney was compensating. This resulted in a total decline in GFR. GFR for the induced group was significantly higher compared to the model and non-induced group (*P* < 0.01). There was no significant difference (*P* = 0.976) in GFR between the model and non-induced group. Thus, renal function in the induced group was significantly better compared to the model and non-induced group; however, this improvement did not restore full function as compared with the normal group (*P* < 0.01) (Figure 9).

### 7: TGF-β1 immunohistochemistry assay

**Figure 10:**
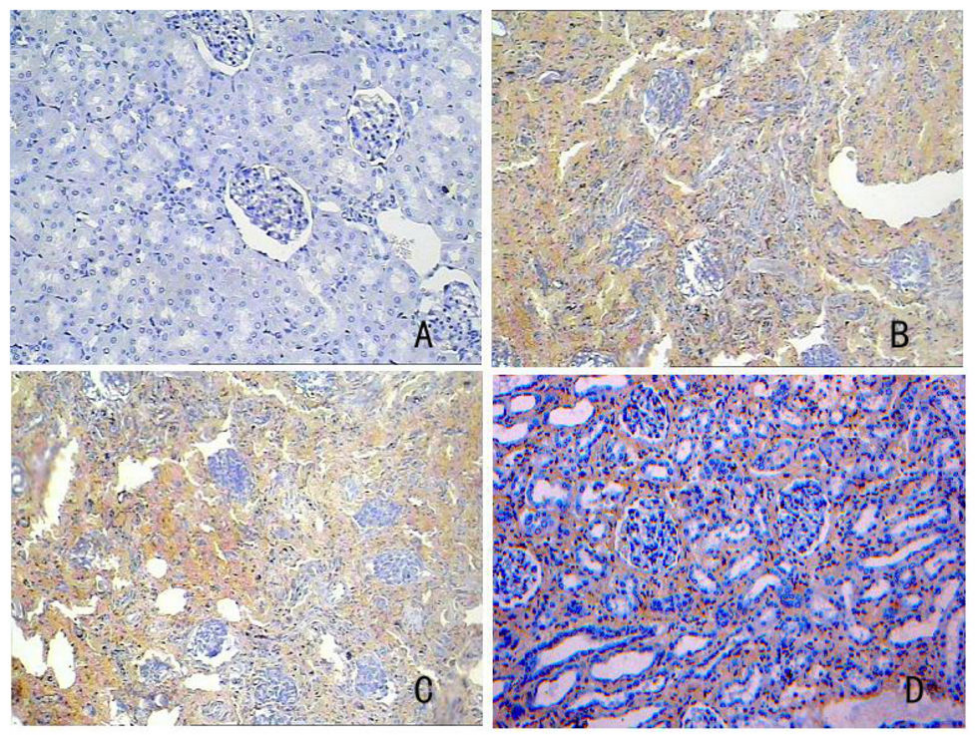
Immunohistochemical expression of TGF-β1. A. Normal group. B. Model group. C. Non-induced group. D. Induced group.

The results of the TGF-β1 immunohistochemistry assay are illustrated in Table 4 and Figure 10. Twenty non-overlapping fields at 200X magnification were calculated in each case. The ratio of positive staining area within the field of vision to total tubulointerstitial area (after removal of the tubular lumen space) was calculated and averaged. A one-way ANOVA was used for statistical analysis because the data have only one factor (group). The results of a one-way ANOVA revealed a significant difference among experimental groups (*P* < 0.01) According to multiple comparisons, there was a difference between certain pairs of groups. The positive staining in the induced group was statistically lower than that in the model or non-induced group (*P* < 0.01).

**Table 4:** Immunohistochemical expression results of TGF-β1.

**Groups**	**Fields**	**TGF-β1 expression**
Normal group	20	1.58 ± 0.15*
Model group	20	27.7 ± 1.0
Non-induced group	20	26.7 ± 0.6*
Induced group	20	16.4 ± 1.4*

The results of one-way ANOVA indicated *P < 0.01 when values were compared between any two groups except between model and non-induced group.

### 8: Distribution of transplanted cells in left renal tissue

**Figure 11:**
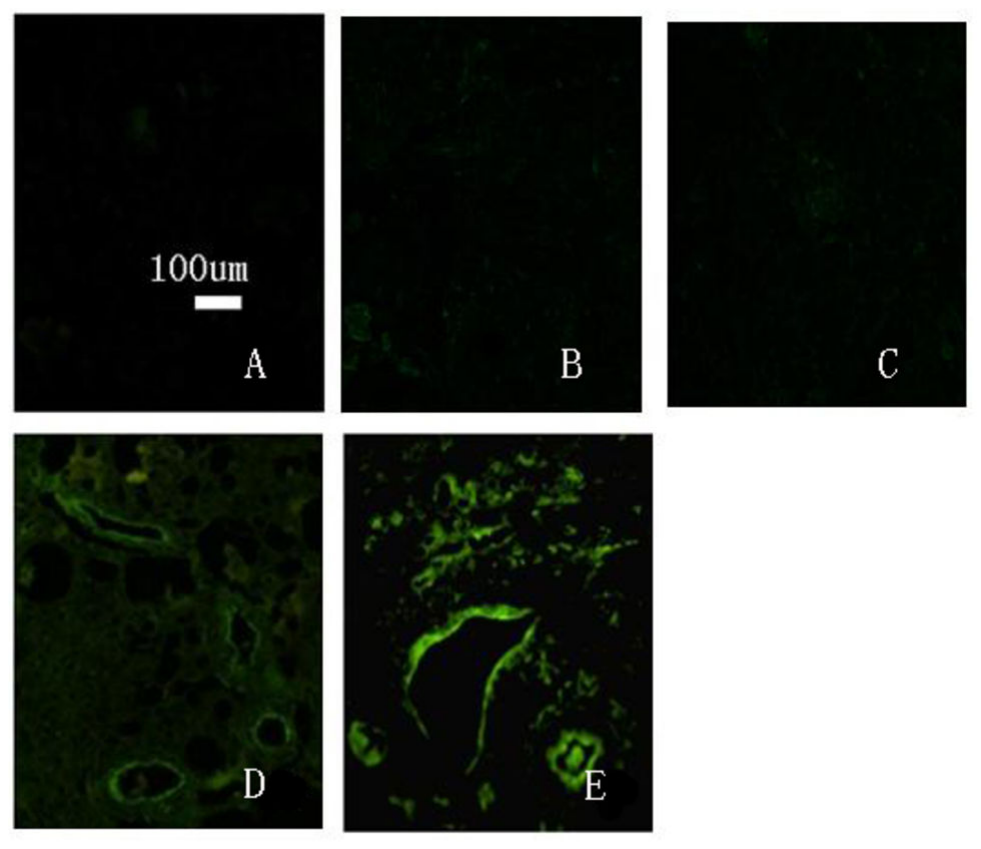
Distribution of GFP-labeled cells in left kidney tissues (× 100). A. Normal group. B. Model group. C. Non-induced group. D and E. Induced group in the left kidney tissues and fluorescent cell distribution, showing accumulation in renal tubules, glomerular capillary loops and small arteries.

**Figure 12:**
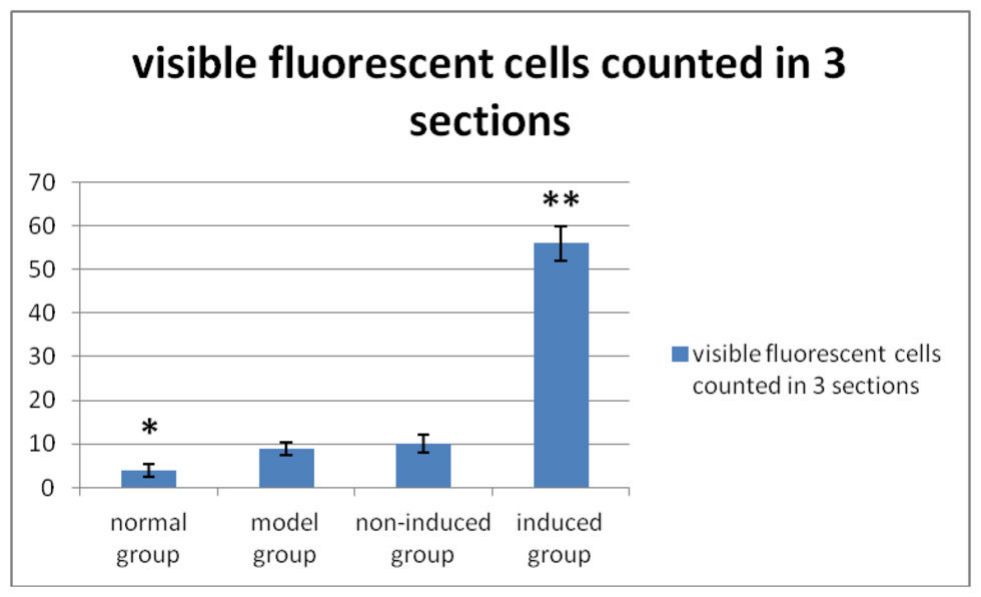
Visible fluorescent cells were counted in three sections.

The distribution of transplanted cells was observed in conventional, 5-µm paraffin sections of renal tissue after dewaxing under a fluorescence microscope (Figure 11). The results showed that left renal tissue from all rabbits of the induced group featured GFP-labeled green fluorescent cells. There were no GFP-positive cells in the left kidneys from the normal, model, or non-induced groups. This may be due to the inability of cells from the non-induced group to implant in renal tissue, and are thus rejected. At 8 weeks after transplantation, visible fluorescent cells were abundant in the renal tubules, glomerular capillary loop and small arteries. Visible fluorescent cells were counted in three sections. A one-way ANOVA was used for statistical analysis because the data have only one factor (group) (Figure 12). The results of a one-way ANOVA revealed significant differences between experimental groups (*P* < 0.01). It showed *P* < 0.05 when the normal group compared to the other three groups, and *P* < 0.01 when the induced group compared to the other three groups. The induced group has the most fluorescent cells.The normal group has the least fluorescent cells.

Results are means ± SD (n = 3). A one-way ANOVA revealed significant differences among groups (*P* < 0.01).*The normal group compared to the other three groups (*P* < 0.05). **The induced group compared to the other three groups (*P* < 0.01). The induced group has the most fluorescent cells.The normal group has the least fluorescent cells.

## Discussion

Stem cells exhibit infinite proliferative capacity and can differentiate into multiple potential cell types [14,15]. They are not only used for studying mechanisms of development and aging, but can also be used to study genetic diseases and to develop treatments for clinically refractory diseases and organ function reconstruction. Stem cell research is at the frontier of life science research [16]. Due to insufficient supply of stem cells, however, the development of methods and procedures using stem cells are seriously restricted. Stem cells obtained from adult tissue are limited in number and the differentiation potential is relatively low. There are ethical concerns with the use of stem cells from embryonic sources and there are potential problems with immune rejection and poor control of differentiation and proliferation *in vivo*. Therefore, a search for new sources of stem cells is one of the major areas of emphasis in stem cell research.

The present study used a natural inducer of stem cells, and then infused the induced cells back into rabbits that had undergone treatment to model RIF. The ability of the transplanted stem cells to protect from, or prevent renal injury was observed. The results showed that transplantation of induced stem cells produced decreases in SCr and BUN values. SPECT showed rebounding of bilateral GFR. Renal biopsy showed the distribution of kidney cells labeled with GFP. All the evidence demonstrated that the inducer (from our laboratory) [13] stimulated proliferation of autologous fibroblasts into multipotent cells, and that treatment of RIF rabbits had a positive therapeutic effect.

RIF is due to a variety of primary or secondary disease processes, leading to glomerular sclerosis, renal fibrosis, and replacement of normal tissue structure by deposition of extracellular matrix, ultimately causing irreversible damage [17,18]. Prognosis of kidney disease is closely related to the degree of involvement of renal fibrosis. Renal tubular fibrosis is a reflection of the decline in kidney function and the degree of disease severity, making it an important index of prognosis. Therefore, the study of tubulointerstitial fibrosis for assessing chronic renal failure is relevant. The unilateral ureter ligation model is the most widely used experimental animal model for RIF [6]. Unilateral ureter ligation causes obstruction in the renal drainage system, increases in urinary pressure, reduces renal blood flow, obstructs venous drainage, promotes infiltration of mesangial cells, increases proliferation of fibroblasts, induces scar formation, and leads to acute renal functional changes and chronic kidney structural damage, which all simulate the clinical form of ureteral obstruction that leads to renal interstitial injury [19]. Animals in this model did not have high blood pressure, albuminuria, and dyslipidemia or compromised immune function. Thus, this model is suitable for the study of renal fibrosis and related cellular factors. The model was found to exhibit an increase in size of the obstructed kidney, cortical thinning, visible infiltration of renal interstitial lymphatic mononuclear cells (as seen by light microscopy), renal tubular expansion, epithelial cell degeneration, atrophy, necrosis, increased interstitial area, and fibrosis. The infiltration of inflammatory cells may be a property of the disease model itself. With increasing time of obstruction, the severity of the lesions progressively worsened, validating the success of this model.

Numerous studies have demonstrated the role of TGF-β1 is the strongest one kind of cytokine to induce fibrosis, and its overexpression can lead to glomerulosclerosis and renal interstitial fibrosis [20,21,22]. So we detected the TGF-β1 expression in renal tissues of each group. The positive staining in the induced group was statistically lower than that in the model or non-induced group (*P* < 0.01). It showed after cell transplantation the RIF were improved in induced group.

The present study demonstrated that eight weeks after transplantation of induced or un-induced skin cells, both SCr and BUN were reduced in the induced group as compared to the un-induced group. Further decreases were observed at 12 weeks and 16 weeks after establishment of the model. Renal biopsy of visible renal tubules from the induced group showed moderate atrophy, basement membrane thickening, tubular multifocal expansion, glomerular capsule cavity expansion, scattered infiltration of renal interstitial inflammatory cells, and mild fibrotic changes. The numbers of inflammatory cells in the induced group were less than that in the non-induced and model group, suggesting that the cell transplantation may be beneficial for kidney function. SPECT analysis showed that total GFR in the normal rabbit group was 92.6 ml/min, in the non-induced group was 51.8 ml/min, and that in the induced group was 67.1 ml/min. The results demonstrated that transplantation of induced autologous stem cells promoted partial restoration of renal structure and function after fibrotic injury. The possible mechanism for this protective effect of induced autologous stem cells may involve their differentiation into mature renal parenchymal cells and/or destruction of the damaged mature cells so as to achieve restoration of structure and function.

This study has two innovative aspects. First, most investigators currently use rats or mice to model RIF, whereas we chose the rabbit. The rabbit model has biochemical indexes and pathogenesis that are more similar to humans and is easier to use than other animal models. Second, the current cell therapy for kidney disease uses bone marrow hematopoietic stem cells as the source of the bone marrow stromal cells, but does not yet use induced autologous stem cell transplantation.

Use of induced autologous stem cells has the following advantages: 1) It avoids the ethical concerns involved in use of embryonic stem cells; 2) it solves the problem of there being a limited supply of stem cells; 3) it avoids the use of foreign sources of cells that may cause immune rejection and poor control [11,23,24]; 4) the procedure involves injecting the cells directly from femoral artery to the left renal artery, giving the cells direct access to kidney lesions, which improves the utilization rate of the cells; and 5) the procedure involves routine monitoring of SCr and BUN to evaluate kidney function, and SPECT for more complete evaluation of kidney function.

## Conclusions

The rabbit model of RIF was successfully established. Transplantation of induced autologous stem cells can repair kidney damage. The repair occurred by both inhibition of further development of RIF and partial reversal of pre-existing RIF. The induced autologous stem cells showed beneficial effects, including improvement of renal function.

## References

[1] Lin F, Cordes K, Li L, Hood L, Couser WG et al. (2003) Hematopoietic stem cells contribute to the regeneration of renal tubules after renal ischemia-reperfusion injury in mice. J Am Soc Nephrol 14: 1188-1199. doi:10.1097/01.ASN.0000061595.28546.A0. PubMed: 12707389. 12707389

[2] Rookmaaker MB, Smits AM, Tolboom H, Van 't Wout K, Martens AC et al. (2003) Bone-marrow-derived cells contribute to glomerular endothelial repair in experimental glomerulonephritis. Am J Pathol 163: 553-562. doi:10.1016/S0002-9440(10)63683-8. PubMed: 12875975. 12875975 PMC1868209

[3] Prodromidi EI, Poulsom R, Jeffery R, Roufosse CA, Pollard PJ et al. (2006) Bone marrow-derived cells contribute to podocyte regeneration and amelioration of renal disease in a mouse model of Alport syndrome. Stem Cells 24: 2448-2455. doi:10.1634/stemcells.2006-0201. PubMed: 16873763. 16873763

[4] Kale S, Karihaloo A, Clark PR, Kashgarian M, Krause DS et al. (2003) Bone marrow stem cells contribute to repair of the ischemically injured renal tubule. J Clin Invest 112: 42-49. doi:10.1172/JCI200317856. PubMed: 12824456. 12824456 PMC162291

[5] Docherty NG, O'Sullivan OE, Healy DA, Fitzpatrick JM, Watson RW (2006) Evidence that inhibition of tubular cell apoptosis protects against renal damage and development of fibrosis following ureteric obstruction. Am J Physiol Renal Physiol 290: F4-13. PubMed: 16339963. 16339963 10.1152/ajprenal.00045.2005

[6] Klahr S, Morrissey J (2003) Obstructive nephropathy and renal fibrosis: The role of bone morphogenic protein-7 and hepatocyte growth factor. Kidney Int Suppl: S105-S112. PubMed: 14531782. 10.1046/j.1523-1755.64.s87.16.x 14531782

[7] Carrion FA, Figueroa FE (2011) Mesenchymal stem cells for the treatment of systemic lupus erythematosus: is the cure for connective tissue diseases within connective tissue? Stem Cell Res Ther 2: 23. doi:10.1186/scrt64. PubMed: 21586107. 21586107 PMC3152993

[8] Takahashi K, Yamanaka S (2006) Induction of pluripotent stem cells from mouse embryonic and adult fibroblast cultures by defined factors. Cell 126: 663-676. doi:10.1016/j.cell.2006.07.024. PubMed: 16904174. 16904174

[9] Huangfu D, Maehr R, Guo W, Eijkelenboom A, Snitow M et al. (2008) Induction of pluripotent stem cells by defined factors is greatly improved by small-molecule compounds. Nat Biotechnol 26: 795-797. doi:10.1038/nbt1418. PubMed: 18568017. 18568017 PMC6334647

[10] Park IH, Zhao R, West JA, Yabuuchi A, Huo H et al. (2008) Reprogramming of human somatic cells to pluripotency with defined factors. Nature 451: 141-146. doi:10.1038/nature06534. PubMed: 18157115. 18157115

[11] Nishikawa S, Goldstein RA, Nierras CR (2008) The promise of human induced pluripotent stem cells for research and therapy. Nat Rev Mol Cell Biol 9: 725-729. doi:10.1038/nrm2466. PubMed: 18698329. 18698329

[12] Kunter U, Rong S, Djuric Z, Boor P, Müller-Newen G et al. (2006) Transplanted mesenchymal stem cells accelerate glomerular healing in experimental glomerulonephritis. J Am Soc Nephrol 17: 2202-2212. doi:10.1681/ASN.2005080815. PubMed: 16790513. 16790513

[13] Zhu XQ, Pan XH, Wang W, Chen Q, Pang RQ et al. (2010) Transient in vitro epigenetic reprogramming of skin fibroblasts into multipotent cells. Biomaterials 31: 2779-2787. doi:10.1016/j.biomaterials.2009.12.027. PubMed: 20044135. 20044135 PMC2909839

[14] Morigi M, Imberti B, Zoja C, Corna D, Tomasoni S et al. (2004) Mesenchymal stem cells are renotropic, helping to repair the kidney and improve function in acute renal failure. J Am Soc Nephrol 15: 1794-1804. doi:10.1097/01.ASN.0000128974.07460.34. PubMed: 15213267. 15213267

[15] Kunter U, Rong S, Boor P, Eitner F, Müller-Newen G et al. (2007) Mesenchymal stem cells prevent progressive experimental renal failure but maldifferentiate into glomerular adipocytes. J Am Soc Nephrol 18: 1754-1764. doi:10.1681/ASN.2007010044. PubMed: 17460140. 17460140

[16] Uchimura H, Marumo T, Takase O, Kawachi H, Shimizu F et al. (2005) Intrarenal injection of bone marrow-derived angiogenic cells reduces endothelial injury and mesangial cell activation in experimental glomerulonephritis. J Am Soc Nephrol 16: 997-1004. doi:10.1681/ASN.2004050367. PubMed: 15744001. 15744001

[17] Li J, Campanale NV, Liang RJ, Deane JA, Bertram JF et al. (2006) Inhibition of p38 mitogen-activated protein kinase and transforming growth factor-beta1/Smad signaling pathways modulates the development of fibrosis in adriamycin-induced nephropathy. Am J Pathol 169: 1527-1540. doi:10.2353/ajpath.2006.060169. PubMed: 17071578. 17071578 PMC1780196

[18] Hewitson TD (2009) Renal tubulointerstitial fibrosis: common but never simple. Am J Physiol Renal Physiol 296: F1239-F1244. doi:10.1152/ajprenal.90521.2008. PubMed: 19144691. 19144691

[19] Cochrane AL, Kett MM, Samuel CS, Campanale NV, Anderson WP et al. (2005) Renal structural and functional repair in a mouse model of reversal of ureteral obstruction. J Am Soc Nephrol 16: 3623-3630. doi:10.1681/ASN.2004090771. PubMed: 16221872. 16221872

[20] Misseri R, Rink RC, Meldrum DR, Meldrum KK (2004) Inflammatory mediators and growth factors in obstructive renal injury. J Surg Res 119: 149-159. doi:10.1016/j.jss.2004.02.016. PubMed: 15145697. 15145697

[21] Verrecchia F, Mauviel A (2002) Transforming growth factor-beta signaling through the Smad pathway: role in extracellular matrix gene expression and regulation. J Invest Dermatol 118: 211-215. doi:10.1046/j.1523-1747.2002.01641.x. PubMed: 11841535. 11841535

[22] Wang W, Koka V, Lan HY (2005) Transforming growth factor-beta and Smad signalling in kidney diseases. Nephrology (Carlton) 10: 48-56. doi:10.1111/j.1440-1797.2005.00334.x. PubMed: 15705182. 15705182

[23] Lewitzky M, Yamanaka S (2007) Reprogramming somatic cells towards pluripotency by defined factors. Curr Opin Biotechnol 18: 467-473. doi:10.1016/j.copbio.2007.09.007. PubMed: 18024106. 18024106

[24] Jaenisch R, Young R (2008) Stem cells, the molecular circuitry of pluripotency and nuclear reprogramming. Cell 132: 567-582. doi:10.1016/j.cell.2008.01.015. PubMed: 18295576. 18295576 PMC4142810

